# Quantitative susceptibility mapping of basal ganglia iron is associated with cognitive and motor functions that distinguish spinocerebellar ataxia type 6 and type 3

**DOI:** 10.3389/fnins.2022.919765

**Published:** 2022-08-18

**Authors:** Cherie L. Marvel, Lin Chen, Michelle R. Joyce, Owen P. Morgan, Katherine G. Iannuzzelli, Stephen M. LaConte, Jonathan M. Lisinski, Liana S. Rosenthal, Xu Li

**Affiliations:** ^1^Department of Neurology, Johns Hopkins University School of Medicine, Baltimore, MD, United States; ^2^F.M. Kirby Research Center for Functional Brain Imaging, Kennedy Krieger Institute, Baltimore, MD, United States; ^3^Department of Radiology and Radiological Science, Johns Hopkins University School of Medicine, Baltimore, MD, United States; ^4^Fralin Biomedical Research Institute at VTC, Virginia Tech, Roanoke, VA, United States; ^5^Biomedical Engineering and Mechanics, Virginia Tech, Blacksburg, VA, United States

**Keywords:** SCA3, SCA6, QSM = quantitative susceptibility mapping, cerebellum, cerebellar dentate, cognition, motor, MRI

## Abstract

**Background:**

In spinocerebellar ataxia type 3 (SCA3), volume loss has been reported in the basal ganglia, an iron-rich brain region, but iron content has not been examined. Recent studies have reported that patients with SCA6 have markedly decreased iron content in the cerebellar dentate, coupled with severe volume loss. Changing brain iron levels can disrupt cognitive and motor functions, yet this has not been examined in the SCAs, a disease in which iron-rich regions are affected.

**Methods:**

In the present study, we used quantitative susceptibility mapping (QSM) to measure tissue magnetic susceptibility (indicating iron concentration), structural volume, and normalized susceptibility mass (indicating iron content) in the cerebellar dentate and basal ganglia in people with SCA3 (*n* = 10) and SCA6 (*n* = 6) and healthy controls (*n* = 9). Data were acquired using a 7T Philips MRI scanner. Supplemental measures assessed motor, cognitive, and mood domains.

**Results:**

Putamen volume was lower in both SCA groups relative to controls, replicating prior findings. Dentate susceptibility mass and volume in SCA6 was lower than in SCA3 or controls, also replicating prior findings. The novel finding was that higher basal ganglia susceptibility mass in SCA6 correlated with lower cognitive performance and greater motor impairment, an association that was not observed in SCA3. Cerebellar dentate susceptibility mass, however, had the opposite relationship with cognition and motor function in SCA6, suggesting that, as dentate iron is depleted, it relocated to the basal ganglia, which contributed to cognitive and motor decline. By contrast, basal ganglia volume loss, rather than iron content, appeared to drive changes in motor function in SCA3.

**Conclusion:**

The associations of higher basal ganglia iron with lower motor and cognitive function in SCA6 but not in SCA3 suggest the potential for using brain iron deposition profiles beyond the cerebellar dentate to assess disease states within the cerebellar ataxias. Moreover, the role of the basal ganglia deserves greater attention as a contributor to pathologic and phenotypic changes associated with SCA.

## Introduction

Iron is involved in a number of important functions of the central nervous system, such as myelin production and neurotransmitter synthesis (Zecca et al., 2004; Ward et al., 2014). However, disruption to local brain iron concentrations and changes in iron deposition can lead to impairments of motor function and cognition (Ward et al., 2014; Du et al., 2016; Ayton et al., 2017; Chen L. et al., 2021). Moreover, in neurodegenerative diseases, changes in iron homeostasis often correlate with disease severity, suggesting that iron deposition is a marker for (and possibly contributor to) neuropathological states (Dominguez et al., 2015; He et al., 2015; Wisnieff et al., 2015; Harrison et al., 2016; Van Bergen et al., 2016, 2018; Ayton et al., 2019). If iron deposition in neurodegenerative diseases could be measured and tracked over time, disease expression and progression could be characterized, which could assist in disease prognosis.

Measuring brain iron levels *in vivo* using MRI has been facilitated by the recent developments of quantitative susceptibility mapping (QSM) techniques, which allow direct mapping of tissue magnetic susceptibility by solving the field-to-susceptibility dipole deconvolution using MR phase measurements (Liu et al., 2009, 2013, 2015; Shmueli et al., 2009; Wharton and Bowtell, 2010; Li et al., 2011, 2015; Schweser et al., 2012; Deistung et al., 2013, 2017; Bilgic et al., 2014; Haacke et al., 2015; Langkammer et al., 2015; Wang and Liu, 2015; Wei et al., 2015; Bao et al., 2016) aging (SWI) and phase imaging (Haacke et al., 2004, 2005; Duyn et al., 2007), QSM overcomes several limitations including blooming artifacts and non-local contributions from neighboring voxels (Liu et al., 2015). Recent studies have shown that QSM-based tissue susceptibility measures correlate well with histochemically-measured tissue iron concentration (Liu et al., 2009; Schweser et al., 2011; Langkammer et al., 2012; Lim et al., 2013; Zheng et al., 2013), with high sensitivity and specificity to tissue iron changes in most gray matter regions (Langkammer et al., 2012; Lim et al., 2013; Zheng et al., 2013). In addition, because QSM provides excellent contrast to delineate iron-rich deep nuclei, QSM can also be used to acquire enhanced tissue volume measurements in these structures (Lim et al., 2013; Cobzas et al., 2015; Hanspach et al., 2017; He et al., 2017; Zhang et al., 2018; Li et al., 2019).

Spinocerebellar ataxia (SCA) is a genetic and progressive neurodegenerative disease in which cell death occurs in the cerebellum, leading to degradative changes in movement, cognition, and mood (Kansal et al., 2017; Hoche et al., 2018; Slapik et al., 2019; Kronemer et al., 2021; Morgan et al., 2021; NINDS, 2021; Joyce et al., 2022). Research has only begun to examine iron deposition related to SCA and other forms of ataxia. A 2-year longitudinal investigation in Friedrich’s ataxia (FA), which is a non-SCA form of ataxia, measured iron concentration in the cerebellar dentate twice using QSM, with measures 2 years apart (Ward et al., 2019). Results indicated that cerebellar dentate iron concentration was greater, and volume was smaller, than that of controls at baseline. Two years later, however, iron concentration in the dentate rose more in FA while dentate structural volume loss had similar rate in FA as compared to controls, suggesting that structural volume loss in the dentate preceded iron accumulation. More recently, Deistung et al. (2022) reported QSM measure of dentate iron concentration and volume in SCA types 1, 2, 3, and 6 and healthy controls in a cross-sectional design (Deistung et al., 2022). They reported higher dentate iron concentrations in SCA1 and SCA2 compared to that of controls, with no change in SCA3. By contrast, SCA6 showed lower dentate iron concentration than in controls. All groups showed smaller dentate volumes compared to controls, and this difference was greatest in SCA6 and smallest in SCA3. In the SCA6 patients only, the ataxia severity scores negatively correlated with dentate iron concentration and volume. Taken together, these studies suggest that cerebellar ataxia types differ in their presentation of cerebellar atrophy and dentate iron deposition.

While limited research has been conducted regarding iron deposition in SCA, more is known regarding the brain volume changes in this disease. Atrophy typically involves the cerebellum in SCA, but may also include extra-cerebellar regions, depending upon the subtype (Seidel et al., 2012). For example, in SCA3, pathology can extend into the nearby pons (Rüb et al., 2002, 2003a,^2004^, ^2005^, ^2006^), midbrain (Rüb et al., 2006), and basal ganglia (BG) (Bürk et al., 1996; Dürr et al., 1996, Rüb et al., 2003b; Lukas et al., 2008). By comparison, in SCA6, pathology is thought to be largely circumscribed to the cerebellum (Ishikawa et al., 1999), with some evidence for less severe neurodegeneration (relative to SCA3) in the pons (Gierga et al., 2009; Reetz et al., 2013), midbrain (Gomez et al., 1997; Gierga et al., 2009), and BG (Gomez et al., 1997).

Pathology of the BG may be particularly relevant to SCA and other cerebellar disorders. The cerebellum and BG are two subcortical brain regions that are anatomically interconnected via thalamic projections (Hoshi et al., 2005; Bostan and Strick, 2018). Pathway mapping in non-human primates has demonstrated connections from the cerebellar dentate to the thalamus (TH) to the putamen (PT). Conversely, a separate pathway travels from the subthalamic nucleus (STN) to the pontine nucleus to the cerebellar cortex. Besides their anatomical connections, the two sub-cortical brain regions are also functionally similar and have a special working relationship. The cerebellum is primarily involved in fine timing and coordination of movements (Holmes, 1939). The BG is important for initiating motor sequences (Jin et al., 2014) and for grouping sequential actions into larger “chunks” (Wymbs et al., 2012). Given their similarities and interconnectedness, it is not surprising that the BG and cerebellum work together during motor and cognitive processes (Doya, 2000; Bostan and Strick, 2018). It is possible that the cerebellum modulates BG activity based on internal models or error signals, as it does with the motor system for voluntary movements (Hoshi et al., 2005).

Like the cerebellar dentate, the BG nuclei are also rich in iron, with the brain’s highest (densest, μg/g) iron levels found in the BG sub-regions (highest to lowest): PT, globus pallidus (GP), and caudate nuclei (CN) as in one post-mortem study (Ramos et al., 2014). By comparison, iron levels were lower in the cerebellar dentate. In SCA, there is evidence to suggest that BG subregions lose volume following cerebellar volume loss. A 2-year longitudinal study that included SCA1, SCA3, and SCA6 participants examined cerebellar and BG structure (Reetz et al., 2013). They observed a significant decline in BG volume (caudate and putamen) in each group across 2 years, compared to controls. Importantly, cerebellar volume, while smaller than that of controls, remained unchanged during this same time period. Thus, BG progressively lost volume *after* cerebellar structural volume loss had plateaued.

In the present study, 7T QSM MRI was used to measure tissue magnetic susceptibility (indicating iron concentration), structural volume and normalized susceptibility mass (indicating iron content) of the cerebellar dentate, BG, and other iron-rich subcortical nuclei in participants with SCA3 or SCA6 and healthy controls. The aim of this study was to assess abnormal brain iron distribution and structural volume loss associated with SCA3 and SCA6 and examine how these measures associated with changes of motor function, cognition, and mood.

## Materials and methods

### Subjects

Individuals with SCA3 (*n* = 10; age in years *M* = 51.1, *SD* = 16.4), SCA6 (*n* = 6; age in years *M* = 64.4, *SD* = 7.53), and healthy controls (*n* = 9; age in years *M* = 47.6, *SD* = 14.6) were recruited for this study. Duration of illness was available via chart review for all ataxia participants. The number of CAG repeats were available for nine SCA3 and five SCA6 participants (see Table 1). Medications for the SCA3 and SCA6 groups consisted of psychotropics (30 and 50%, respectively) and muscle relaxants (30 and 16.7%, respectively) (see Supplementary Table 1 for a full list of medications by group).

**TABLE 1:** Participant demographics.

	Healthy Controls (*n* = 9)	SCA3 (*n* = 10)	SCA6 (*n* = 6)	*P*-value
Age (years)	47.5 (14.6) [36.3, 58.8] *W* = 0.939, *p* = 0.569	51.09 (16.4) [39.4, 62.8], *W* = 0.772, ***p* = 0.007**	64.4 (7.53) [56.5, 72.3], *W* = 0.951, *p* = 0.752	0.051^∧^
Gender M:F	3:6	2:8	3:3	0.458
Education (years)	16.7 (3.32) [14.1, 19.2] *W* = 872, *p* = 0.128	16.5 (2.37) [14.8,18.2] *W* = 0.936, *p* = 0.507	17.2 (1.84) [15.2,19.1] *W* = 0.692, ***p* = 0.005**	0.799^∧^
Duration of Illness (years)	–	10.39 (3.50) [7.89, 12.89] *W* = 0.931, *p* = 0.462	8.76 (4.53) [4.00, 13.5] *W* = 0.969, *p* = 0.883	0.43
Number of CAG repeats	–	69.22 (5.85) (*n* = 9) [64.5, 73.7] *W* = 0.944, *p* = 0.626	22.00 (0.00) (*n* = 5) [22.0, 22.0] *W* = null (no range)	**<0.001^∧^**

SD, standard deviation; M, male; F, female. Data are presented as mean (standard deviation) [95% CI lower limit, upper limit], Shapiro-Wilk test of normality (W), and its corresponding p-value. For three-group comparisons, the initial p-value indicates omnibus ANOVA test results, and subsequent values indicate pairwise post hoc p-values. A benign number of CAG repeats in the ATXN3 gene implicated in SCA3 is < 45; a benign number of CAG repeats in the CACNA1A gene implicated in SCA6 is < 19 (Mayo_Clinic_Laboratories, 1995–2022).

^∧^Non-parametric tests were used; boldface indicates significance.

Inclusion criteria consisted of participants’ being: (1) between the ages of 18–80 years of age, (2) at least 10 years of education, (3) right-handed, (4) native English speaker, and (5) for the SCA participants, genetically confirmed with SCA3 or SCA6. Participants were excluded for the following criteria: (1) illicit substance use within 60 days of the MRI scans, (2) history of Axis I psychiatric disorders (including alcohol or drug dependence), (3) neurological disorders (aside from SCA3 or SCA6), (4) severe or unstable medical disorder, (5) history of head injury with loss of consciousness for greater than 5 min, or (6) any condition that would rule out an MRI (e.g., metal within the body, pregnancy, claustrophobia).

This study was approved by Johns Hopkins Medicine Institutional Review Board. All participants provided written informed consent to participate in this study.

### MRI image acquisition

All scans were performed on an ultra-high field 7T MRI scanner (Achieva; Philips Healthcare, Best, the Netherlands) with a 32-channel head coil (Nova Medical). Structural images were collected using a sagittal T1-weighted magnetization prepared gradient-echo (MPRAGE) sequence with 1.0 mm isotropic resolution: repetition time (TR) = 5.0 ms; echo time (TE) = 1.88 ms; flip angle = 7 degrees; TFE factor = 352; shot interval = 4,500 ms; inversion delay = 563 ms; SENSE factor = 2 × 2; field of view (FOV) = 220 × 220 × 180 mm^3^; with reconstruction matrix size = 224 × 224 × 180; total scan time = 2′15′′. High-resolution QSM data were acquired using a 3D multi-echo gradient echo (GRE) sequence with 0.7 mm isotropic resolution: TE1/ΔTE/TR = 5/5/28 ms with 5 monopolar echoes; band width = 403.7 Hz/pix; flip angle = 12 degrees; axial slab; FOV = 252 × 210 × 126 mm^3^ with reconstruction matrix size = 384 × 384 × 180; SENSE factor = 2 × 2; total scan time = 8′49′′.

### MRI processing

Quantitative susceptibility maps were generated using a pipeline as in a previous study (Chen L. et al., 2021) using the Johns Hopkins University/Kennedy Krieger Institute QSM Toolbox (version 3.0).^1^ MR phase images acquired with the 3D GRE sequence were first unwrapped using a best-path based method (Abdul-Rahman et al., 2005). A brain mask was then obtained using FSL BET (Smith, 2002) on the GRE magnitude image at the first echo with 1 voxel erosion. Background fields were eliminated using the VSHARP method with a maximum kernel size of 8 mm and a regularization parameter of 0.05 (Schweser et al., 2011; Wu et al., 2012b). The resulting local frequency shift maps were then averaged over echoes to obtain a higher signal-to-noise (SNR) reconstruction (Wu et al., 2012a). QSM dipole inversion was conducted with the structural feature-based collaborative reconstruction with automatic referencing to central cerebrospinal fluid (CSF) regions (SFCR+0) method (Bao et al., 2016; Chen L. et al., 2021).

**FIGURE 1:**
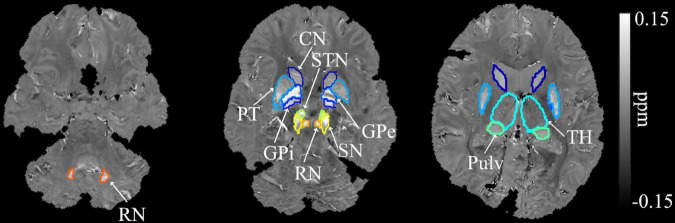
Example segmentation of the dentate nuclei, basal ganglia, and related iron-rich structures overlaid on the QSM image of a SCA6 participant. DN, dentate nucleus; CN, caudate nucleus; PT, putamen; GPi, globus pallidus internal; GPe, globus pallidus external; RN, red nucleus; SN, substantia nigra; STN, subthalamic nucleus; Pulv, pulvinar; TH, thalamus.

The procedures for segmenting the regions of interest (ROIs) were as follows. First, the T_1_-weighted MPRAGE (T_1_W) image was co-registered to the GRE magnitude image at the first echo with affine transformation using Advanced Normalization Tools (ANTs) (Avants et al., 2011). The co-registered T_1_W image was then skull-stripped by applying the same brain mask obtained from the QSM processing described above. The co-registered T_1_W and QSM images were then segmented using a QSM/T_1_ multi-atlas matching approach, which was developed as part of the Johns Hopkins University brain atlases (Li et al., 2019) and freely available on MRIcloud (mricloud.org). From the obtained brain parcellation map, nine bilateral deep gray matter regions were extracted, including the dentate nuclei (DN), basal ganglia [BG: CN, globus pallidus internal (Gpi), globus pallidus external (Gpe), PT, STN, substantia nigra (SN)], pulvinar (Pulv), and red nucleus (RN) (Figure 1). The thalamus (TH), excluding pulvinar, was also extracted for comparison. The ROI masks were further eroded with 1 voxel to avoid possible partial volume effects and used to calculate the mean magnetic susceptibility value in each ROI as a measures of tissue iron concentration. The volume of each structure was determined by multiplying the number of voxels in each ROI by the voxel size. The whole brain volume was calculated similarly based on the brain mask used (i.e., multiplying the number of voxels within the mask by the voxel size). To compensate for the effect of different brain sizes across participants, individual structural volumes were normalized as follows: Corrected ROI Volume = Original Structure Volume × (Group Mean whole brain Volume/Subject whole brain Volume).

Since cerebellar atrophy is a known pathological feature in SCA3 and SCA6 that may lead to increased tissue iron concentration and increased magnetic susceptibility values, a measure reflecting normalized total iron content in each brain region was derived as normalized susceptibility mass = mean magnetic susceptibility x Corrected ROI Volume. Such susceptibility mass measure was used as the major measure of regional iron deposition or iron loss. Note that the susceptibility mass measure was not calculated for the thalamus ROI due to its myelin-induced negative QSM values when referenced to CSF.

### Supplemental assessments

The following tests were given to both ataxic patients and healthy controls, excluding the International Cooperative Ataxia Rating Scale, which was only administered to ataxia participants.

#### Montreal cognitive assessment

The Montreal Cognitive Assessment (MOCA) version 7.1 is a cognitive screening tool that assesses deficits within the following domains: visuospatial/executive, naming, memory, attention, language, abstraction, delayed recall, and orientation (Nasreddine et al., 2005).

#### Reading subtest of the wide range achievement test

The Wide Range Achievement Test (WRAT-3) reading subtest is used to estimate premorbid verbal intelligence (Wilkinson, 1993). One healthy control was not administered the WRAT-3 assessment because they were highly familiar with the test.

#### International cooperative ataxia rating scale

The International Cooperative Ataxia Rating Scale (ICARS) is an ataxia rating scale that assesses the level of impairments as a result of ataxia within the following areas: postural and gait disturbances, limb ataxia, dysarthria, and oculomotor dysfunction (Trouillas et al., 1997).

#### Finger tapping task

Participants were administered a locally developed finger tapping task (Eierud et al., 2011; Morgan et al., 2021). Participants were asked to tap in synchrony with a cross that flashed at 1, 2, 3, or 4 Hz and to rest otherwise. Visual feedback was provided as a vertical slider bar that indicated whether tapping was slower than the target speed (to the left of the cross) or faster than target speed (to the right of the cross). Participants received a practice session consisting of two 20 s blocks at 1 and 2 Hz. The full task was then administered, which consisted of 32 tapping blocks at 1, 2, 3, or 4 Hz, lasting 16, 18, or 20 s in duration. Rest blocks, lasting 26, 28, or 30 s, alternated with the tapping blocks. Only one target frequency was presented per block. The order of block-frequency was generated pseudo-randomly with the constraints that each frequency was presented eight times, and that the task began with 20 s of rest, followed by a tapping block at 1 Hz. The total task lasted 25 min. Variables of interest were tapping speed, measured as inter-tap intervals or seconds/tap, and tapping accuracy, measured as root-mean-squared error (RMSE) relative to target. For simplicity, results reporting focused on 1 and 4 Hz only.

#### Hamilton anxiety rating scale

The Hamilton Anxiety Rating Scale (HAM-A) is a rating scale that quantifies the severity of current anxiety symptoms (Hamilton, 1959).

#### Hamilton depression rating scale

The Hamilton Depression Rating Scale (HAM-D) is a rating scale that quantifies the severity of current depression symptoms (Hamilton, 1960).

### Statistical analysis

Statistics were performed using IBM SPSS Statistics, Macintosh, version 27.0 (IBM Corp., Armonk, NY, United States), with the exception of non-parametric correlations. For these, the “corr” function in MATLAB 2021b (MathWorks, Natick, Mass) was used for Spearman’s rank correlations with the exact permutation distributions with an adjust for ties for small sample size. All statistics were performed using two-tailed statistical analyses with an alpha level of *p* < 0.05.

#### Demographic variables

To assess differences in demographic variables across all three groups (healthy controls, SCA3, and SCA6), one-way analysis of variance (ANOVA) tests were used for the following continuous variables: age, education, MOCA, WRAT-3, ICARS (only in SCAs), HAM-A, HAM-D, and finger tapping at 1 and 4 Hz RMSE. The following assessments were comprised of multiple ordinal datasets, which were added together, and the sum total scores were treated as continuous data: MOCA, ICARS, HAM-A, and HAM-D. To compare gender, a Pearson Chi-Square test was used. To assess the duration of illness and number of CAG repeats between SCA3 and SCA6, independent samples *t*-tests were used. The Levene’s Test for Equality of Variances was used to identify unequal variances within an ANOVA test, and Shapiro-Wilk tests were used to determine if continuous variables followed a non-normal distribution. If unequal variances or non-normal distributions were observed, then Kruskal-Wallis H tests were used instead of one-way ANOVAs, and Mann-Whitney tests were used instead of independent samples *t*-tests for between-group comparisons.

#### Regions of interest volume, susceptibility mass, and magnetic susceptibility across groups

One-way ANOVAs were used to assess the difference across all three groups for volume, susceptibility mass, and magnetic susceptibility values within ROIs. As described above, data were tested for unequal variances and non-normal distributions using a Levene’s Test and Shapiro-Wilk test, respectively. If unequal variances or non-normal distributions were observed, then a Kruskal-Wallis *H*-test was used instead of an ANOVA. Following the group comparison, if a main effect was observed, independent samples *t*-tests or Mann-Whitney tests (for non-normal distributions) were used for between-group *post hoc* comparisons.

#### Regions of interest volume, susceptibility mass, and magnetic susceptibility associations among the regions of interests

To examine associations among the ROIs, groups were examined separately. ROI volumes were correlated to each other using Pearson’s correlations, or Spearman’s rank correlations for data sets that contained a non-normal distribution. Susceptibility mass and magnetic susceptibility values were similarly correlated across ROIs.

#### Regions of interest volume, susceptibility mass, and magnetic susceptibility associations with supplemental variables

ROI volumes were correlated with supplemental test scores per group using Pearson correlations for the MOCA and ICARS (total sum scores), WRAT, RMSE of finger tapping at 1 and 4 Hz, HAM-A, and HAM-D. Exploratory analyses were also conducted using scores from the sub-scales within the MOCA and ICARS to examine whether particular cognitive domains or motor functions were associated with ROI volumes. When sub-scales were used in the MOCA and ICARS, e.g., MOCA Language, or when distribution was non-normal, Spearman’s rank correlations were used instead. Susceptibility mass and magnetic susceptibility values were similarly correlated with the supplemental test scores.

#### Susceptibility mass values and aging

The effect of aging on susceptibility mass associations between cerebellar dentate and BG was examined by correlating susceptibility mass values between the dentate and BG regions, per group, controlling for age. Spearman’s rank correlations were used for all comparisons due the lack of homogeneity of variance within the cerebellar dentate values.

## Results

Groups did not significantly differ on the demographic variables of age, gender, and education. Duration of illness did not differ between the SCA3 and SCA6 groups. The number of CAG repeats was higher in SCA3 than in SCA6, which was expected (Table 1). When neurological and cognitive assessments were compared across groups (Table 2), the healthy control group scored higher than did both patient groups on the MOCA. Groups did not differ on the WRAT-3, finger tapping at 1 and 4 Hz, or the mood scales (HAM-A, HAM-D); patient groups did not differ on the ICARS.

**TABLE 2:** Cognitive, neurological, and mood assessment scores.

	Healthy controls (*n* = 9)	SCA3 (*n* = 10)	SCA6 (*n* = 6)	*P*-value
MOCA total	28.6 (1.42) [27.5, 30.0] *W* = 0.899, *p* = 0.246	26.8 (1.69) [25.6, 28.0] *W* = 0.876, *p* = 0.118	24.5 (4.51) [19.8, 29.2] *W* = 0.821, *p* = 0.089	0.027, 0.**026^A–B^, 0.026^∧*A*–*C*^, 0.**635^∧*B*–*C*^
WRAT-3	49.3 (3.45) (*n* = 8) [46.4, 52.1] *W* = 0.931, *p* = 0.529	46.4 (3.50) [43.9, 48.9] *W* = 0.965, *p* = 0.839	49.2 (1.94) [47.1, 51.2] *W* = 0.812, *p* = 0.452	0.127
ICARS total	–	39.5 (12.8) [30.3, 48.7] *W* = 0.934, *p* = 0.491	35.7 (16.5) [18.3, 53.0] *W* = 0.919, *p* = 0.501	0.611
Finger tapping 1 Hz RMSE	0.315 (0.138) [0.209, 0.421] *W* = 0.836, *p* = 0.052	0.345 (0.089) [0.281, 0.409] *W* = 0.956, *p* = 0.735	0.332 (0.070) [0.259, 0.405] *W* = 0.831, *p* = 0.109	0.834
Finger tapping 4 Hz RMSE	0.092 (0.055) [0.050, 0.134] *W* = 0.796, ***p* = 0.018**	0.145 (0.076) [0.091, 0.200] *W* = 0.851, *p* = 0.060	0.155 (0.115) [0.034, 0.275] *W* = 0.879, *p* = 0.266	0.239^∧^
HAM-A	4.67 (2.83) [2.49, 6.84] *W* = 0.950, *p* = 0.695	10.2 (6.56) [5.51, 14.9] *W* = 0.952, *p* = 0.695	5.67 (5.16) [0.250, 11.1] *W* = 0.889, *p* = 0.311	0.068
HAM-D	1.78 (1.64) [0.520, 3.04] *W* = 0.874, *p* = 0.137	5.20 (4.47) [2.00, 8.40] *W* = 0.895, *p* = 0.194	3.67 (3.14) [0.370, 6.96] *W* = 0.941, *p* = 0.664	0.111

RMSE, root mean squared error. Data are presented as mean (standard deviation) [95% CI lower limit, upper limit], Shapiro-Wilk test of normality (W), and its corresponding p-value. For three-group comparisons, the initial p-value indicates omnibus ANOVA test results, and subsequent values indicate pairwise post hoc p-values. A = Healthy controls, B = SCA3, C = SCA6.

^Non-parametric tests were used; boldface indicates significance.

**FIGURE 2:**
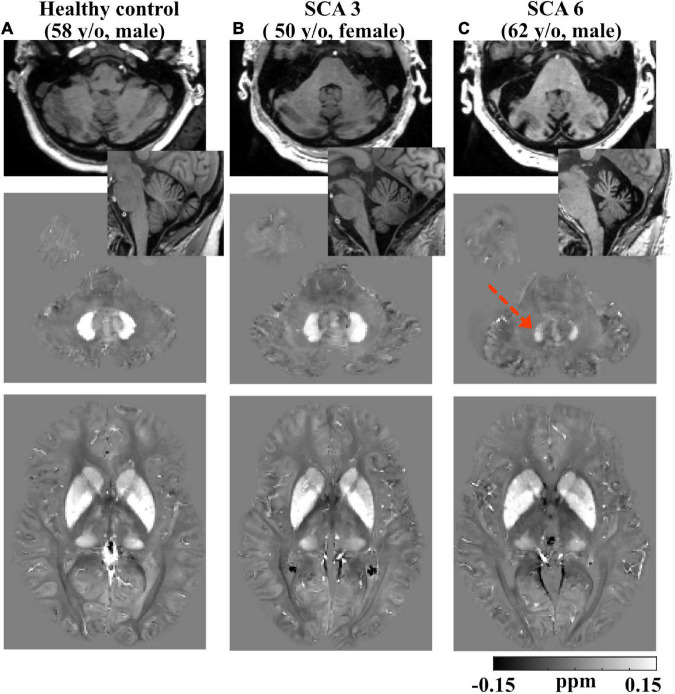
Example T1-weighted and QSM images. Row 1: axial and sagittal T1-weighted images showing smaller total cerebellar volume in a (62 y/o, M) SCA6 subject **(C)** vs. a (50 y/o, F) SCA3 subject **(B)** and a (58 y/o, M) healthy control subject **(A)**; slight atrophy in cerebellum was also observed in SCA3. Row 2: axial QSM images showing the dentate. Dotted arrow denotes lower QSM and atrophied dentate in the SCA6 subject. Row 3: axial QSM showing the basal ganglia. Gray scales in QSM images are in the range of (–0.15, 0.15) ppm. All the images are rigidly transformed to MNI space for better comparison of the same anatomical structures.

Example anatomical T_1_W images of the cerebellum in both axial and sagittal views are shown for a healthy control (58 y/o, M) participant in Figure 2, in comparison to one SCA3 (50 y/o, F) and one SCA6 (62 y/o, M) participant. In addition, QSM images of the iron-rich cerebellar dentate and BG regions of the same participants are also shown. Cerebellar atrophy including the cortex and dentate are clearly visible in the SCA6 participant.

**FIGURE 3:**
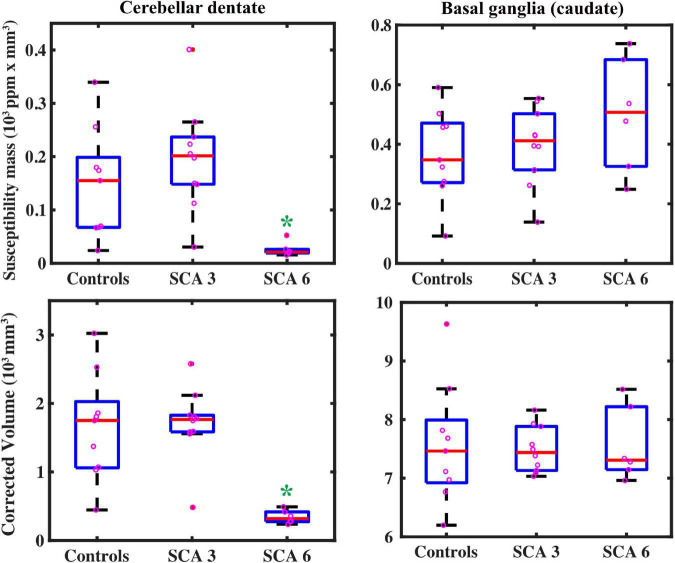
QSM-derived normalized susceptibility mass (mean magnetic susceptibility × Corrected ROI Volume in the unit of 10^3^ ppm × mm^3^) in the dentate nuclei (top left) showed significant group differences. * = SCA6 < SCA3 (*p* < 0.001) and SCA6 < healthy controls (*p* = 0.002). Group differences were not significant in the basal ganglia (using caudate as a proxy, top right) SCA6 = SCA3 = healthy controls (*p* = 0.26). Volume measures showed similar results: in the dentate (bottom left), * = SCA6 < SCA3 (*p* < 0.001) and SCA6 < healthy controls (*p* = 0.001); groups did not differ for caudate volume (bottom right), SCA6 = SCA3 = healthy controls (*p* = 0.96), but SCA6 = SCA3 < healthy controls in the putamen (*p* = 0.046, see Table 3). Boxplots represent first (Q1) and third (Q3) quartiles; bars indicate minimum (Q0) and maximum (Q4) values, excluding outliers; red lines represent the median.

Groups were statistically compared on measures of volume, susceptibility mass, and magnetic susceptibility. However, because no corrections were applied for multiple comparisons, these analyses were considered exploratory. Cerebellar dentate volume was reduced in the SCA6 group relative to the SCA3 and control groups (Table 3 and Figure 3). Both SCA groups showed reduced volume in the PT relative to that of controls. The SCA6 group showed markedly lower susceptibility mass values in the cerebellar dentate relative to the SCA3 and control groups (Table 4 and Figure 3). In terms of tissue iron concentration, indicated by magnetic susceptibility, dentate iron concentration was lower in the SCA6 group than in the SCA3 group. The SCA3 group showed higher values in the SN relative to that of the SCA6 and healthy control groups (Supplementary Table 2).

**TABLE 3:** Corrected ROI volumes (in the unit of 10^3^ mm^3^) compared across groups.

Corrected ROI volume (SD)				
ROI	Healthy controls (*n* = 9)	SCA3 (*n* = 10)	SCA6 (*n* = 6)	*P*-value
Caudate	7.57 (1.02) [6.79, 8.36] *W* = 0.949, *p* = 0.676	7.49 (0.392) [7.21, 7.77] *W* = 0.925, *p* = 0.403	7.58 (0.634) [6.91, 8.24] *W* = 0.850, *p* = 0.157	0.961
GPi	1.27 (0.153) [1.15, 1.39] *W* = 0.925, *p* = 0.435	1.04 (0.279) [0.842, 1.24] *W* = 0.939, *p* = 0.540	1.25 (0.218) [1.02, 1.47] *W* = 0.981, *p* = 0.954	0.079
GPe	3.81 (0.470) [3.45, 4.17] *W* = 0.961, *p* = 0.810	3.36 (0.820) [2.77, 3.95] *W* = 0.875, *p* = 0.115	3.63 (0.772) [2.82, 4.44] *W* = 0.954, *p* = 0.771	0.390
Putamen	8.66 (1.17) [7.76, 9.56] *W* = 0.947, *p* = 0.660	7.61 (0.977) [6.92, 8.32] *W* = 0.945, *p* = 0.615	7.54 (0.484) [7.03, 8.05] *W* = 0.878, *p* = 0.259	**0.046, 0.048^*A*–*B*^, 0.046^*A*–*C*^, 0.**869^*B*–*C*^
Thalamus	9.04 (0.916) [8.84, 9.75] *W* = 0.816, ***p* = 0.031**	9.08 (0.829) [8.49, 9.68] *W* = 0.930, *p* = 0.446	9.76 (0.909) [8.81, 10.71] *W* = 0.808, *p* = 0.070	0.182^∧^
Pulvinar	2.61 (.472) [2.24, 2.97] *W* = 0.930, *p* = 0.484	2.26 (0.686) [1.77, 2.75] *W* = 0.851, *p* = 0.059	2.19 (0.430) [1.74, 2.64] *W* = 0.945, *p* = 0.704	0.289
STN	0.350 (0.107) [0.268, 0.432] *W* = 0.935, *p* = 0.531	0.271 (0.067) [0.224, 0.319] *W* = 0.984, *p* = 0.984	0.281 (0.044) [0.235, 0.327] *W* = 0.889, *p* = 0.313	0.100
SN	1.29 (0.274) [1.08, 1.50] *W* = 0.968, *p* = 0.873	1.28 (0.206) [1.13, 1.43] *W* = 0.955, *p* = 0.730	1.12 (0.185) [0.930, 1.32] *W* = 0.975, *p* = 0.924	0.340
RN	0.535 (0.080) [0.473, 0.597] *W* = 0.964, *p* = 0.835	0.447 (0.096) [0.379, 0.516] *W* = 0.922, *p* = 0.372	0.502 (0.064) [0.435, 0.569] *W* = 0.892, *p* = 0.327	0.094
Dentate	1.65 (0.789) [1.05, 2.26] *W* = 0.972, *p* = 0.911	1.71 (0.528) [1.33, 2.09] *W* = 0.857, *p* = 0.070	0.343 (0.097) [0.241, 0.445] *W* = 0.930, *p* = 0.580	**<0.001, 0.**863^*A*–*B*^, 0.**001^∧*A*–*C*^**, **<0.001^∧*B*–*C*^**

Gpi, globus pallidus internal; Gpe, globus pallidus external; STN, subthalamic nucleus; SN, substantia nigra; RN, red nucleus. Data are presented as mean (standard deviation) [95% CI lower limit, upper limit], Shapiro-Wilk test of normality (W), and its corresponding p-value. For three-group comparisons, the initial p-value indicates omnibus ANOVA test results, and subsequent values indicate pairwise post hoc p-values. A = Healthy controls, B = SCA3, C = SCA6. ^∧^Non-parametric tests were used; boldface indicates significance.

**TABLE 4:** Normalized susceptibility mass (in the unit of 10^3^ ppm × mm^3^) for ROIs compared across groups.

Normalized susceptibility mass (SD)				
ROI	Healthy controls (*n* = 9)	SCA3 (*n* = 10)	SCA6 (*n* = 6)	*P*-value
Caudate	0.368 (0.151) [0.251, 0.484] *W* = 0.971, *p* = 0.902	0.396 (0.130) [0.303, 0.489] *W* = 0.941, *p* = 0.561	0.502 (0.193) [0.300, 0.704] *W* = 0.951, *p* = 0.745	0.256
GPi	0.145 (0.033) [0.120, 0.170] *W* = 0.989, *p* = 0.242	0.125 (0.049) [0.090, 0.160] *W* = 0.946, *p* = 0.627	0.145 (0.043) [0.100, 0.190] *W* = 0.971. *p* = 0.897	0.504
GPe	0.548 (0.115) [0.460, 0.636] *W* = 0.976, *p* = 0.937	0.501 (0.180) [0.372, 0.629] *W* = 0.916, *p* = 0.328	0.548 (0.215) [0.322, 0.773] *W* = 0.955, 0.782	0.795
Putamen	0.555 (0.192) [0.407, 0.703] *W* = 0.934, *p* = 0.517	0.521 (0.208) [0.372, 0.669] *W* = 0.982, *p* = 0.977	0.603 (0.239) [0.353, 854] *W* = 0.967, *p* = 0.869	0.751
Pulvinar	0.120 (0.065) [0.069, 0.170] *W* = 0.933, *p* = 00.506	0.095 (0.058) [0.054, 0.136] *W* = 0.950, 0.674	0.090 (0.066) [0.021, 0.159] *W* = 0.802, 0.061	0.600
STN	0.041 (0.022) [0.024, 0.057] *W* = 0.893, *p* = 0.213	0.034 (0.013) [0.025, 0.043] *W* = 0.944, *p* = 0.601	0.030 (0.008) [0.021, 0.039] *W* = 0.949, *p* = 0.729	0.446
SN	0.150 (0.061) [0.103, 0.197] *W* = 0.976, *p* = 0.944	0.196 (0.055) [0.157, 0.236] *W* = 0.970, *p* = 0.891	0.142 (0.050) [0.089, 0.194] *W* = 0.876, *p* = 0.250	0.117
RN	0.059 (0.023) [0.041, 0.077] *W* = 0.960, *p* = 0.803	0.062 (0.019) [0.049, 0.076] *W* = 0.872, *p* = 0.105	0.061 (0.027) [0.033, 0.089] *W* = 0.833 m *p* = 0.282	0.949
Dentate	0.148 (0.103) [0.069, 0.227] *W* = 0.921, *p* = 0.402	0.197 (0.099) [0.126, 0.268] *W* = 0.962, *p* = 0.811	0.026 (0.013) [0.012, 0.040] *W* = 0.738, ***p* = 0.015**	**0.002^∧^, 0.002^∧*A*–*C*^, 0.**305^*A*–*B*^**, <0.001^∧*B*–*C*^**

Gpi, globus pallidus internal; Gpe, globus pallidus external; STN, subthalamic nucleus; SN, substantia nigra; RN, red nucleus. Data are presented as mean (standard deviation) [95% CI lower limit, upper limit], Shapiro-Wilk test of normality (W), and its corresponding p-value. For three-group comparisons, the initial p-value indicates omnibus ANOVA test results, and subsequent values indicate pairwise post hoc p-values. A = Healthy controls, B = SCA3, C = SCA6. ^∧^Non-parametric tests were used; boldface indicates significance.

### Susceptibility mass associations among the regions of interests

**FIGURE 4:**
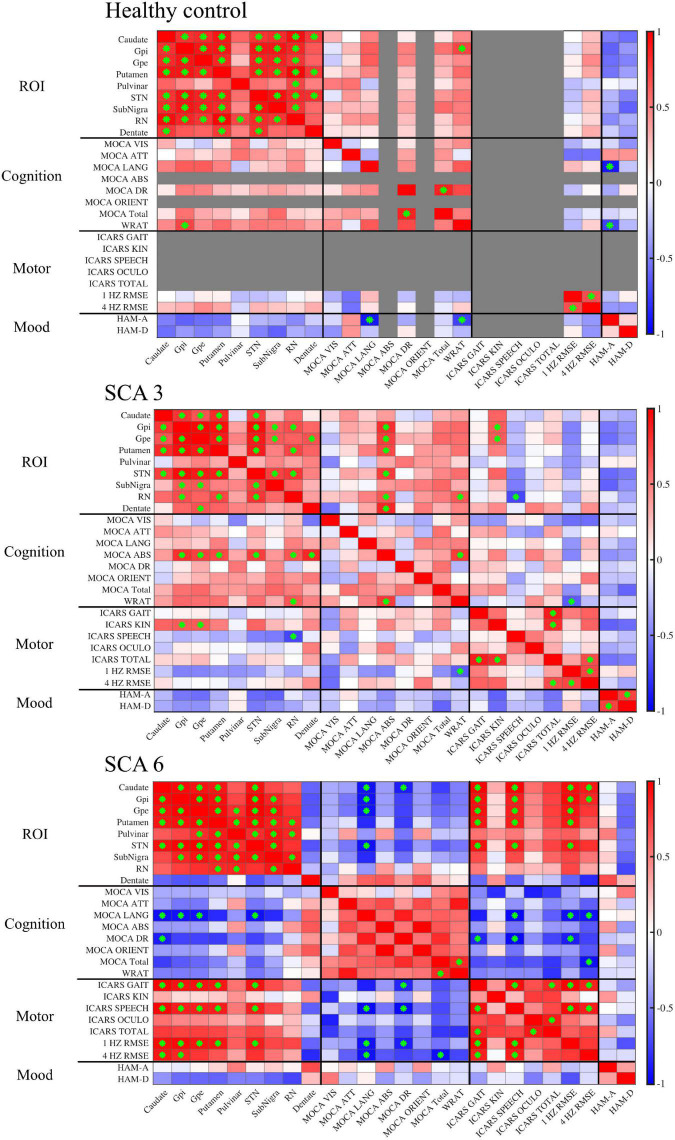
Correlation matrices show associations between QSM-derived normalized susceptibility mass (mean magnetic susceptibility × Corrected ROI Volume in the unit of 10^3^ppm × mm^3^) in the ROIs and cognitive, motor, and mood assessments. Red, positive correlation; blue, negative correlation. Green asterisk indicates *p*-value < 0.05. In controls, scores were grayed out when there was no measurement (ICARS) or no variability across participants (MOCA abstract thinking and orientation). MOCA naming was excluded altogether because there was no score variability for all three groups. Gpi, globus pallidus internal; Gpe, globus pallidus external; STN, subthalamic nucleus; SN, substantia nigra; RN, red nucleus.

Within each studied group, susceptibility mass values within ROIs were correlated to each other showing regional patterns of iron deposition (Figure 4). In controls, much of the ROIs positively correlated with each other, indicating cohesive iron accumulation. In particular, susceptibility mass in the dentate positively correlated with values in the CN, PT, and STN. The SCA3 group followed this general pattern but with less consistency (i.e., less often met statistical threshold) among the ROIs. Unlike in controls, the SCA3 group’s correlations of susceptibility mass involving the dentate were significantly positive with the GPe only. The SCA6 group also followed this general pattern of positive associations among the ROIs, with the marked exception of the cerebellar dentate, in which associations with other ROIs were not significant and actually skewed toward the negative direction. This contrasted with the dentate-related associations observed in the SCA3 and control groups.

### Susceptibility mass associations with supplemental variables

Supplemental test scores were correlated to susceptibility mass in the selected ROIs within each group (Figure 4). A pattern of negative correlations (higher cognitive function at lower susceptibility mass) was revealed in the SCA6 group between the MOCA language sub-scale and susceptibility mass in the CN, GPi, GPe, and STN. It was notable that associations between language scores and susceptibility mass skewed in a positive direction for the dentate, underscoring the inverse relationship between the dentate and other ROIs in SCA6. Delayed recall sub-scale scores negatively correlated with susceptibility mass in the CN. In the SCA3 group, abstract thinking sub-scale scores positively correlated with susceptibility mass in the GPi, GPe, PT, STN, RN, and dentate. Controls did not show correlations between MOCA scores and ROI susceptibility mass values.

For ICARS scores, in the SCA6 group, positive correlations were observed between the gait and speech sub-scales and susceptibility mass in the CN, GPi, GPe, PT, and STN. The SCA3 group revealed a different pattern of results. Kinetic sub-scale scores positively correlated with susceptibility mass in the GPi and GPe; speech sub-scale scores negatively correlated with susceptibility mass in the RN.

The WRAT, RMSE of finger tapping at 1 and 4 Hz, HAM-A, and HAM-D scores were also correlated to susceptibility mass. In the SCA6 group, RMSE of finger tapping positively correlated to susceptibility mass in the CN, GPi, GPe, PT, and STN. In the SCA3 group, WRAT scores positively correlated with susceptibility mass in the RN. Controls showed positive associations between the WRAT and GPi susceptibility mass. Significant associations of susceptibility mass and mood scales and number of CAG repeats (not shown) were not observed.

**FIGURE 5:**
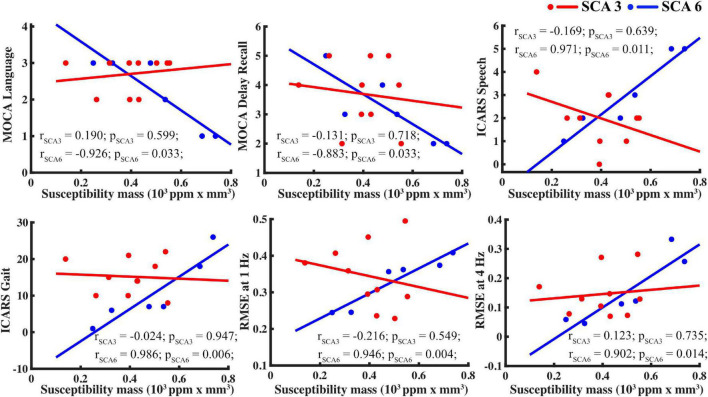
For SCA6 but not SCA3, susceptibility mass in the basal ganglia (using the caudate as proxy) correlated with supplemental measures. Tasks, clockwise from upper left: MOCA language, MOCA delayed recall, ICARS speech, finger tapping RMSE at 4 and 1 Hz, and ICARS gait.

Given the low sample size, scatterplots were visually inspected to ensure that results were not skewed by one or two participants, as shown in Figure 5.

### Impact of magnetic susceptibility and structural volume on associations with susceptibility mass

Because regional iron content (indicated by susceptibility mass) is dependent upon tissue iron levels/concentrations (indicated by magnetic susceptibility) and structural volume, correlations were conducted within each group that examined magnetic susceptibility separately from volume (see Supplementary Figure 1). As expected, correlations between ROIs with respect to magnetic susceptibility and structure volume often tracked together, yet some differences were evident. In the control group, there were notably fewer ROI correlations for volume than for susceptibility. In the SCA3 group, associations among the ROIs differed such that those among the CN, GPi, GPe, and PT were more influenced by susceptibility, whereas those among the STN, SN, and RN were more influenced by volume. Moreover, associations between the ROIs susceptibility mass and motor scores (e.g., ICARS and tapping) were generally volume-driven (comparing Figure 4 and Supplementary Figure 1). In the SCA6 group, associations among the ROIs, as well as between the ROIs susceptibility mass and motor scores and cognitive scores (e.g., MOCA and WRAT), appeared to be driven by susceptibility over volume. Similar to the susceptibility mass analysis (Figure 4), dentate magnetic susceptibility associations presented in the opposite direction from the other ROIs (Supplementary Figure 1A). When dentate susceptibility values were high, cognitive and motor scores skewed toward more favorable outcomes (i.e., higher MOCA scores, lower ICARS scores). The opposite pattern was observed for the BG ROIs, in which higher susceptibility values skewed toward less favorable outcomes (i.e., lower MOCA scores and higher ICARS scores). This relationship supports the iron relocation hypothesis: as iron levels deplete in the dentate and increase in the BG, motor function and cognition becomes impaired in SCA6.

After controlling for age, we found that susceptibility mass in the dentate and BG regions (CN, PT, and STN) positively correlated in controls, skewed toward negative correlations for SCA6, and had no identifiable relationship for SCA3 (Supplementary Table 3).

## Discussion

This study examined iron content levels (as indicated by susceptibility mass) within the iron-rich regions of the cerebellar dentate and BG in people with SCA3 and SCA6. Data revealed several findings that distinguished these two patient groups and underscored the relevance of the basal ganglia in SCA. First, dentate susceptibility mass in SCA6 was markedly reduced relative to those found in the SCA3 and control groups. This finding replicates those from a previous study (Deistung et al., 2022), with significant iron loss in the dentate observed in SCA6, but not in SCA3. Second, susceptibility mass levels in BG regions did not differ across groups, but associations with supplemental measures did. In SCA6, higher BG susceptibility mass was associated with lower performance scores on the MOCA language test, higher ICARS scores for gait and speech, and higher error rates during finger tapping at 1 and 4 Hz. In SCA3, these associations were not found; instead, volume appeared to drive associations with the supplemental measures (i.e., larger volumes with higher MOCA scores and lower ICARS scores). Third, in SCA6, the susceptibility mass levels in the dentate were inversely related to susceptibility mass levels in the BG, as were the associations with supplemental measures. Thus, as iron content levels in the dentate depleted, iron content levels rose in the BG, and this shift was associated with decrements in cognitive and motor function. This relationship was not observed in SCA3.

A positive correlation between the BG and dentate susceptibility mass was observed in healthy controls, which likely reflects a common trend of iron deposition during aging in both regions. It is known that non-heme iron accumulates across brain structures over the life span and may play an important role during brain development, aging, and neurodegeneration (Hallgren and Sourander, 1958; Zecca et al., 2004; Li et al., 2014; Persson et al., 2015; Acosta-Cabronero et al., 2016; Zhang et al., 2018). In the current study, when age was taken into account, susceptibility mass of the cerebellar dentate in healthy controls increased alongside that of the BG. By contrast, susceptibility mass in SCA6 decreased in the dentate while it increased in the BG. In SCA3, the susceptibility mass of these regions was not tied to an identifiable pattern of change. These data suggest that iron depositions take different trajectories in the SCAs than in healthy controls, and there may be heterogeneity of iron deposition patterns between these two SCA subtypes.

The mechanisms underlying changes in brain iron levels are only partially understood. Because iron levels are high within oligodendrocytes, iron levels may increase as oligodendrocytes attempt to repair myelin damage, drawing iron to the region (Bartzokis et al., 2007). Inflammation or neuron damage-induced microgliosis could also increase local tissue iron (Mehta et al., 2013; Thomsen et al., 2015). Alternatively, iron levels may decrease as a simple function of iron-rich cell death. Iron concentration can increase if iron content remains steady, but structural volume is reduced due to loss of other cell types with less or no iron. Thus, measures of both iron content and volume are useful for discerning iron deposition and atrophy-induced increase of tissue iron concentration.

In a recent QSM study of SCA iron levels by Deistung et al. (2022), dentate iron content and volume declined in SCA6, without an increase in iron concentration. This suggested a net loss of iron-rich cells in the dentate. Regions like the BG may have the ability to absorb additional iron that is released into the milieu, which has been considered conceptually as “iron relocation,” observed as inversely associated iron levels between two brain regions (Koeppen et al., 2007; Deistung et al., 2022). Moreover, excess iron can lead to cell death through free radicals (Imam et al., 2017). Thus, an interesting potential consequence in SCA6 may be that iron relocates from the dentate to the BG.

Structural volume measures in this study also revealed group differences. The cerebellar dentate was smaller in the SCA6 group than in the SCA3 or healthy control groups, consistent with a recent study that examined the dentate specifically (Deistung et al., 2022). The PT was smaller in both SCA groups relative to the healthy control group. These findings overlapped with several studies reporting atrophy in the PT in SCA3 and/or SCA6 (Schulz et al., 2010; D’Abreu et al., 2012, Reetz et al., 2013; De Rezende et al., 2015); however, those studies also found atrophy in the CN, which was not observed here. Atrophy in the GP for SCA3 has also been reported (D’Abreu et al., 2012; De Rezende et al., 2015), which was at trend level (*p* = 00.079) in the current study. The sample sizes of SCA3 participants for those studies were larger than in the current study (range: 19–47), which may explain, in part, different results. Our analyses revealed that structural volumes had more influence in SCA3 than iron did, with increased atrophy of the CN, GPi, GPe, PT, and RN correlating with greater severity of motor impairments.

Basal ganglia susceptibility mass in SCA6 correlated with several aspects of speech and language in the ICARS and MOCA assessments. The sample size was small, and these results should be considered tentatively. Yet, current evidence indicates that the cerebellum and BG each play an important role in speech motor control (Cannito and Marquardt, 1997; Ackermann and Hertrich, 2000; Riecker et al., 2005). This aspect of motor function was assessed directly by the ICARS by asking participants to repeat a phonetically challenging phrase and considering other informal verbal communications throughout the exam. The cerebellum and BG also play an important role in language functions that extend beyond frank motor skills, such as working memory and fluency (Chen and Desmond, 2005; Booth et al., 2007; Marvel and Desmond, 2010, 2012; Silveri, 2021). The MOCA language assessment was based on one’s ability to repeat syntactically complex sentences, therefore relying more on comprehension, memory, and fluency than on motor speech. In SCA6, MOCA language scores correlated with ICARS speech scores and finger tapping errors, suggesting that these tasks drew upon similar underlying processes, and elevated iron content influenced this process, namely, in the CN, GPi, GPe, PT, and STN. Interestingly, MOCA and ICARS scores did not correlate in SCA3, which may be indirectly related to a decreased role for iron content in SCA3. Overall, data from this study indicate that the integrity of the BG contributes to speech and language ability in SCA6. This finding is compelling and warrants further investigation.

An obvious limitation of the present study is the relatively small sample size for each SCA group, which may have hampered our ability to discover small effects in terms of brain iron changes, e.g., in the SCA3 group, but our findings were generally consistent with previous studies of larger sample sizes in the same patient populations. Also, the SCA group was on medications that may have impacted performance. However, there are no known effects of these medications on iron deposition. With these limitations in mind, the associations observed between BG iron with motor and cognitive function in SCA6 suggest the potential for using brain iron deposition profiles outside of the cerebellum dentate, with particular attention to the BG, to assess disease states in cerebellar ataxia.

Another potential study limitation was that the selected region of interest for the DN in the present study included the bulk iron-rich region (Figure 1; as also defined in Deistung et al., 2022) instead of the dentate silhouette commonly marked in histology, which could lead to overestimations of the volume and iron content in the DN. However, this should affect all the groups in the same way, so the major conclusions are not expected to change. It should also be noted that QSM measures are not specific to iron and could include contributions from other paramagnetic materials, such as copper or manganese, even though large changes of these are not known to occur in the SCAs. In addition, potential changes in diamagnetic myelin content, e.g., in the thalamus, could also affect QSM measures in those myelin rich regions (Liu et al., 2011; Li et al., 2016); therefore, the potential for thalamic demyelination and atrophy in SCA6 to lead to higher susceptibility and greater motor impairments (as in Supplementary Figure 1) cannot be excluded. Separation of subvoxel paramagnetic and diamagnetic sources, e.g., iron vs. myelin, using more advanced multi-contrast modeling in the future could further help shed light on the underlying pathophysiology (Chen J. J. et al., 2021; Shin et al., 2021).

The results of this study could support or help guide future treatments in SCA. Given the emergence of iron chelating treatments in neurodegenerative diseases (Boddaert et al., 2007; Suzuki et al., 2013), *in vivo* measures of brain iron levels are a logical pre-clinical step toward establishing the feasibility of such treatments in humans. Future studies with larger sample sizes and longitudinal follow-up are warranted.

## Data availability statement

The raw data supporting the conclusions of this article will be made available by the authors, without undue reservation.

## Ethics statement

The studies involving human participants were reviewed and approved by the Johns Hopkins Institutional Review Board. The patients/participants provided their written informed consent to participate in this study.

## Author contributions

CM, KI, OM, SL, and JL performed the material preparation and data collection. LC and XL performed the QSM data processing. CM, LC, MJ, and OM performed the statistical analyses. CM, LC, MJ, and XL wrote the first draft of the manuscript. All authors commented on previous versions of the manuscript, read and approved the final manuscript, and contributed to the study conceptualization and design.

## Footnotes

1
https://godzilla.kennedykrieger.org/QSM/
