# Supplementary material for: Quantitative susceptibility mapping of basal ganglia iron is associated with cognitive and motor functions that distinguish spinocerebellar ataxia type 6 and type 3
Source: Front Neurosci. 2022 Aug 18;16:919765. doi: 10.3389/fnins.2022.919765 (PMC9433989; doi:10.3389/fnins.2022.919765)
Supplement: Supplementary file 1 [file Table_1.pdf]

SupplementTable 1. Relevant medications by group. * Includes: Escitalopram, Mirtazapine, Clozapine, Fluoxetine, Venlafaxine, Amitriptyline; ** Includes: Baclofen, Chlorzoxazone.

**Drug Category**	**Group**		
	**Healthy Controls**	**SCA 3**	**SCA 6**
Psychotropics*	0%	30%	50%
Antiviral (Amantadine)	0%	10%	16.7%
Muscle relaxants**	0%	30%	16.7%
Potassium Channel Blocker (4-Aminopyridine)	0%	10%	0%
GABA agonist (Gapapentin)	0%	10%	0%
Benzothiazole (Riluzole)	0%	10%	0%
No	100%	20%	33%
