# Supplementary material for: Quantitative susceptibility mapping of basal ganglia iron is associated with cognitive and motor functions that distinguish spinocerebellar ataxia type 6 and type 3
Source: Front Neurosci. 2022 Aug 18;16:919765. doi: 10.3389/fnins.2022.919765 (PMC9433989; doi:10.3389/fnins.2022.919765)
Supplement: Supplementary file 2 [file Table_2.pdf]

Supplement Table 2. Magnetic susceptibility values for iron concentration (in the unit of ppm) for ROIs compared across groups. Gpi = globus pallidus internal; Gpe = globus pallidus external; STN = subthalamic nucleus; SN = substantia nigra; RN = red nucleus. Data are presented as mean (standard deviation) [95% CI lower limit, upper limit], Shapiro-Wilk test of normality (W), and its corresponding p-value. For three-group comparisons, the initial p-value indicates omnibus ANOVA test results, and subsequent values indicate pairwise post-hoc p-values. A = Healthy controls, B = SCA3, C = SCA6. ^ = non-parametric tests were used; boldface indicates significance.

**Susceptibility (SD)**				
**ROI**	**Controls**	**SCA 3**	**SCA 6**	**p-value**
Caudate	.048 (.020)<br>[.033, .064]<br>W= .957, p = .764	.053 (.164)<br>[.041, .064]<br>W= .944, p = .601	.065 (.021)<br>[.043, .287]<br>W= .926, p = .551	.265
GPi	.114 (.015)<br>[.102, .125]<br>W= .948, p = .667	.117 (.021)<br>[.102, .132]<br>W= .917, p = .332	.115 (.018)<br>[.096, .134]<br>W= .878, p = .262	.931
GPe	.143 (.019)<br>[.128, .158]<br>W= .910, p = .319	.146 (.023)<br>[.129, .162]<br>W= .815, **p = .022**	.146(.030)<br>[.115, .178]<br>W= .888, p = .309	.592^
Putamen	.065 (.024)<br>[.047, .083]<br>W= .960, p = .799	.067 (.023)<br>[.051, .083]<br>W= .938, p = .530	.079 (.028)<br>[.050, .109]<br>W= .697, p = .869	.527
Thalamus	-.008 (.011)<br>[-.017, .001]<br>W= .953, p = .724	-.011 (.013)<br>[-.020, -.002]<br>W= .854, p = .065	-.018 (.008)<br>[-.026, -.009]<br>W= .924, p = .537	.289
Pulvinar	.044 (.020)<br>[.029, .060]<br>W= .910, p = .317	.037 (.019)<br>[.024, .051]<br>W= .955, p = .725	.0389(.020)<br>[.018, .060]<br>W= .895, p = .347	.741
STN	.110 (.032)<br>[.085, .134]<br>W= .954, p = .728	.122 (.023)<br>[.106, .138]<br>W= .952, p = .696	.106 (.013)<br>[.092, .119]<br>W= .966, p = .861	.380
SN	.113 (.028)<br>[.091, .134]	.151 (.023)<br>[.138, .167]	.123 (.023)<br>[.099, .148]	**.009, .004** $^{\mathbf{A\text{-}B}}$, .472$^{A\text{-}C}$, **.034**$^{\mathbf{B\text{-}C}}$

	W= .966, p = .855	W= .931, p = .460	W= .929, p = .575	
RN	.108 (.034)<br>[.083, .134]<br>W= .920, p = .389	.139 (.032)<br>[.116, .162]<br>W= .959, p = .770	.120 (.044)<br>[.073, .167]<br>W= .897, p = .355	.195
Dentate	.081 (.027)<br>[.061, .102]<br>W= .938, p = .560	.110 (.032)<br>[.086, .133]<br>W= .958, p = .762	.074 (.020)<br>[.053, .094]<br>W= .883, p = .285	**.036,**<br>.058$^{A-B,}$<br>.549$^{A-C,}$<br>**.029** $^{\mathbf{B-C}}$
