# Supplementary material for: Quantitative susceptibility mapping of basal ganglia iron is associated with cognitive and motor functions that distinguish spinocerebellar ataxia type 6 and type 3
Source: Front Neurosci. 2022 Aug 18;16:919765. doi: 10.3389/fnins.2022.919765 (PMC9433989; doi:10.3389/fnins.2022.919765)
Supplement: Supplementary file 3 [file Table_3.pdf]

Supplement Table 3. Correlations of normalized susceptibility mass between the cerebellar dentate and basal ganglia region, controlling for age. Spearman's rank correlations were used due the lack of homogeneity of variance within the cerebellar dentate values.

Group	Caudate	Globus Pallidus internal	Globus Pallidus external	Putamen	Subthalamic nucleus	Substantia nigra
Healthy controls df = 6	**.78, p = .023**	.66, p = .075	.60, p = .12	**.78, p = .021**	**.75, p = .031**	.52, p = .19
SCA3 df = 7	-.23, p = .56	.48, p = .19	.62, p = .08	.49, p = .18	.39, p = .30	.57, p = .11
SCA6 df = 3	-.86, p = .063	-.77, p = .12	-.77, p = .12	-.75, p = .15	-.77, p = .12	-.51, p = .38
