# Supplementary material for: Quantitative susceptibility mapping of basal ganglia iron is associated with cognitive and motor functions that distinguish spinocerebellar ataxia type 6 and type 3
Source: Front Neurosci. 2022 Aug 18;16:919765. doi: 10.3389/fnins.2022.919765 (PMC9433989; doi:10.3389/fnins.2022.919765)
Supplement: Supplementary file 4 [file Image_1.pdf]

Supplement Figure 1

(A) QSM

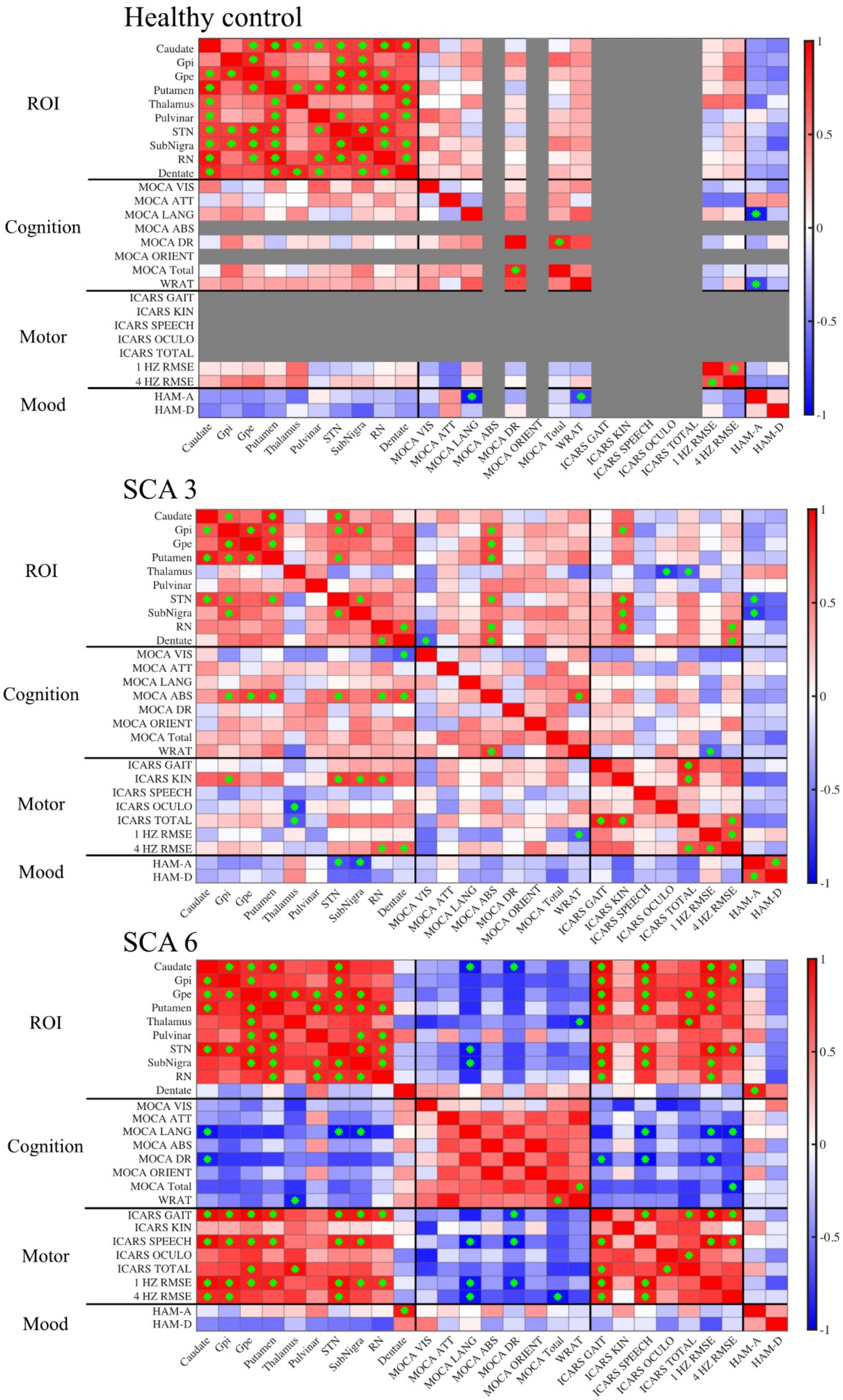

(B) Volume

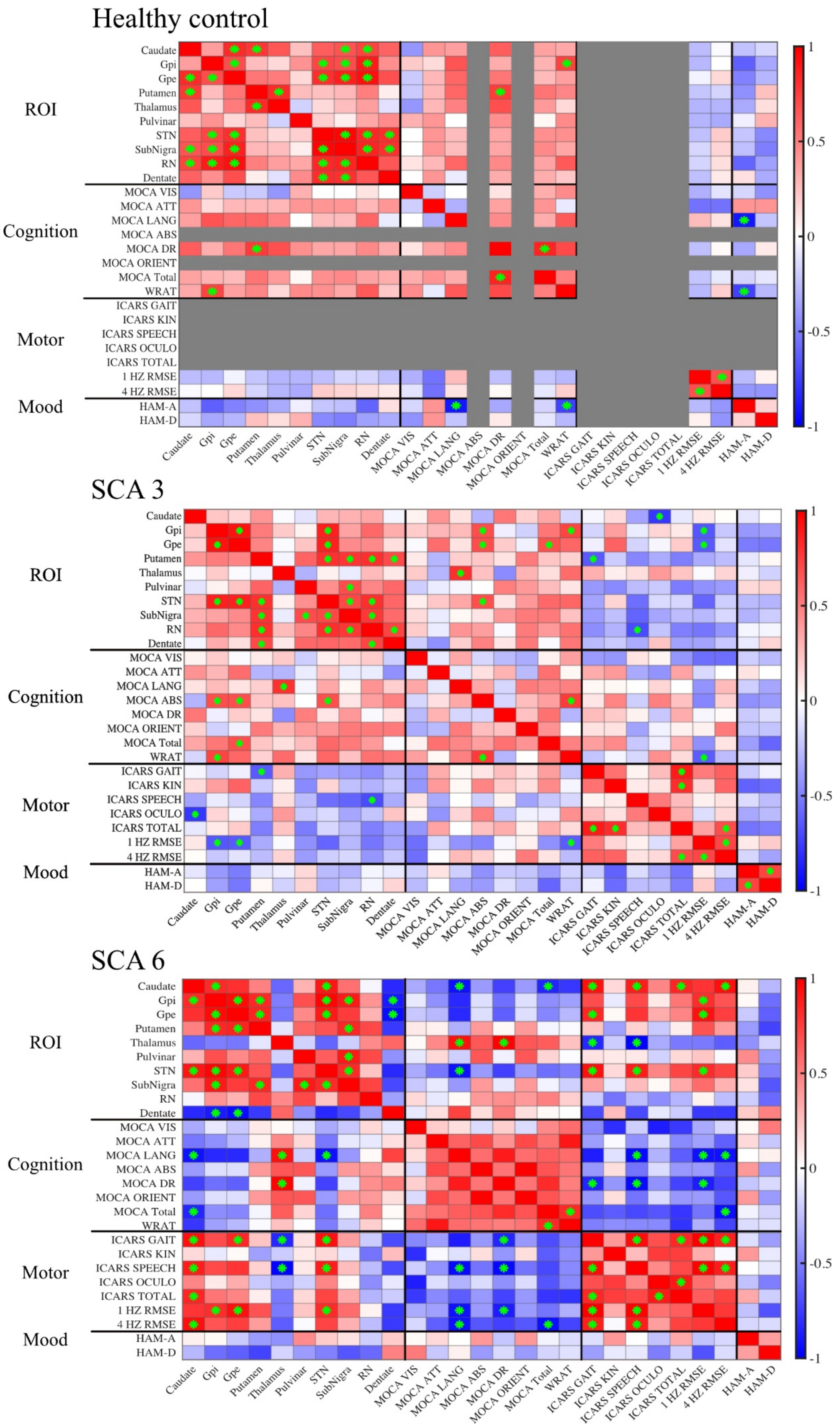
Healthy control
ROI
Cognition
Motor
Mood
Caudate
Gpi
Gpe
Putamen
Thalamus
Pulvinar
STN
SubNigra
RN
Dentate
MOCA VIS
MOCA ATT
MOCA LANG
MOCA ABS
MOCA DR
MOCA ORIENT
MOCA Total
WRAT
ICARS GAIT
ICARS KIN
ICARS SPEECH
ICARS OCULO
ICARS TOTAL
1 HZ RMSE
4 HZ RMSE
HAM-A
HAM-D
1
0.5
0
-0.5
-1
SCA 3
SCA 6

Figure S1. Correlation matrices A and B show associations of QSM-derived susceptibility (A, indicating tissue iron concentration) and structural volumes of the ROIs (B) with cognitive, motor, and mood assessments. Red = positive correlation; blue = negative correlation. Green asterisk indicates p – value < .05. In controls, scores were grayed out when there was no measure (ICARS) or no variability across participants (MOCA abstract thinking and orientation). MOCA naming was excluded altogether because there was no score variability for all three groups. Gpi = globus pallidus internal; Gpe = globus pallidus external; STN = subthalamic nucleus; SN = substantia nigra; RN = red nucleus.
